# Supplementary figures and images for: Serum growth differentiation factor 15 trajectory predicts 28-day mortality in critically ill patients: a multicenter cohort study
Source: PeerJ. 2025 Nov 3;13:e20317. doi: 10.7717/peerj.20317 (PMC12591050; doi:10.7717/peerj.20317)

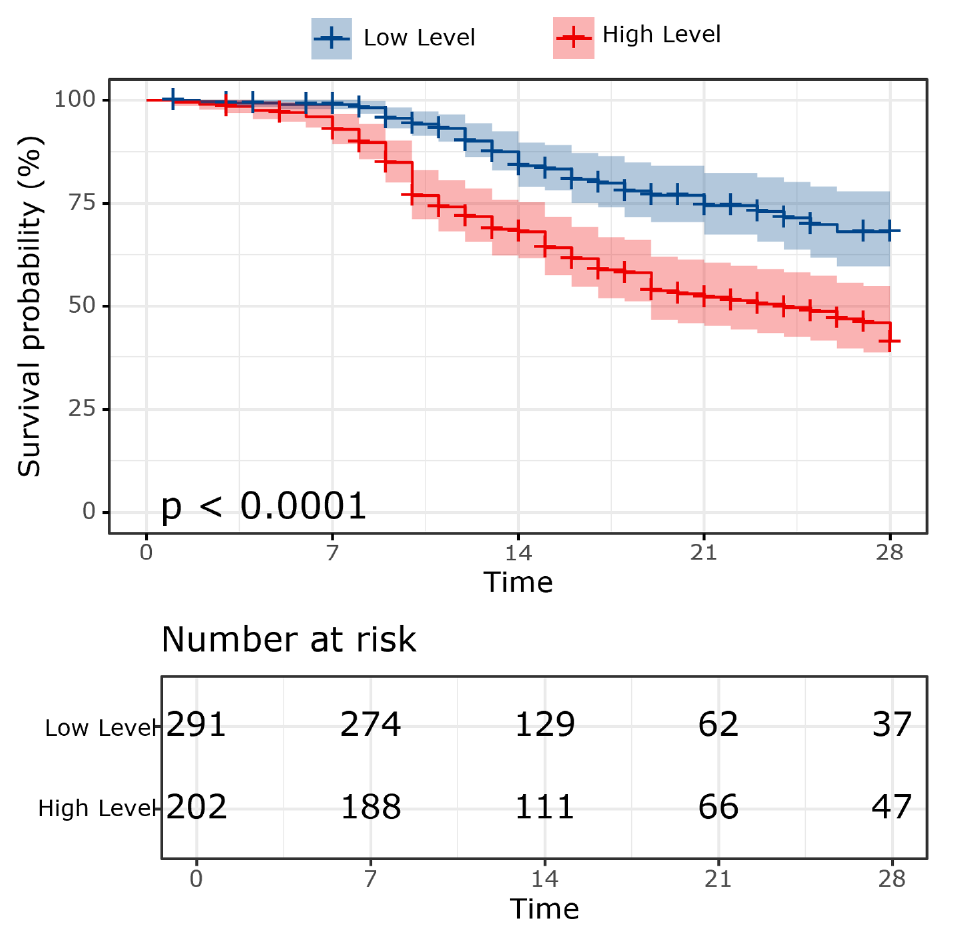

Supplement: Supplemental Information 1 [file peerj-13-20317-s001.png]

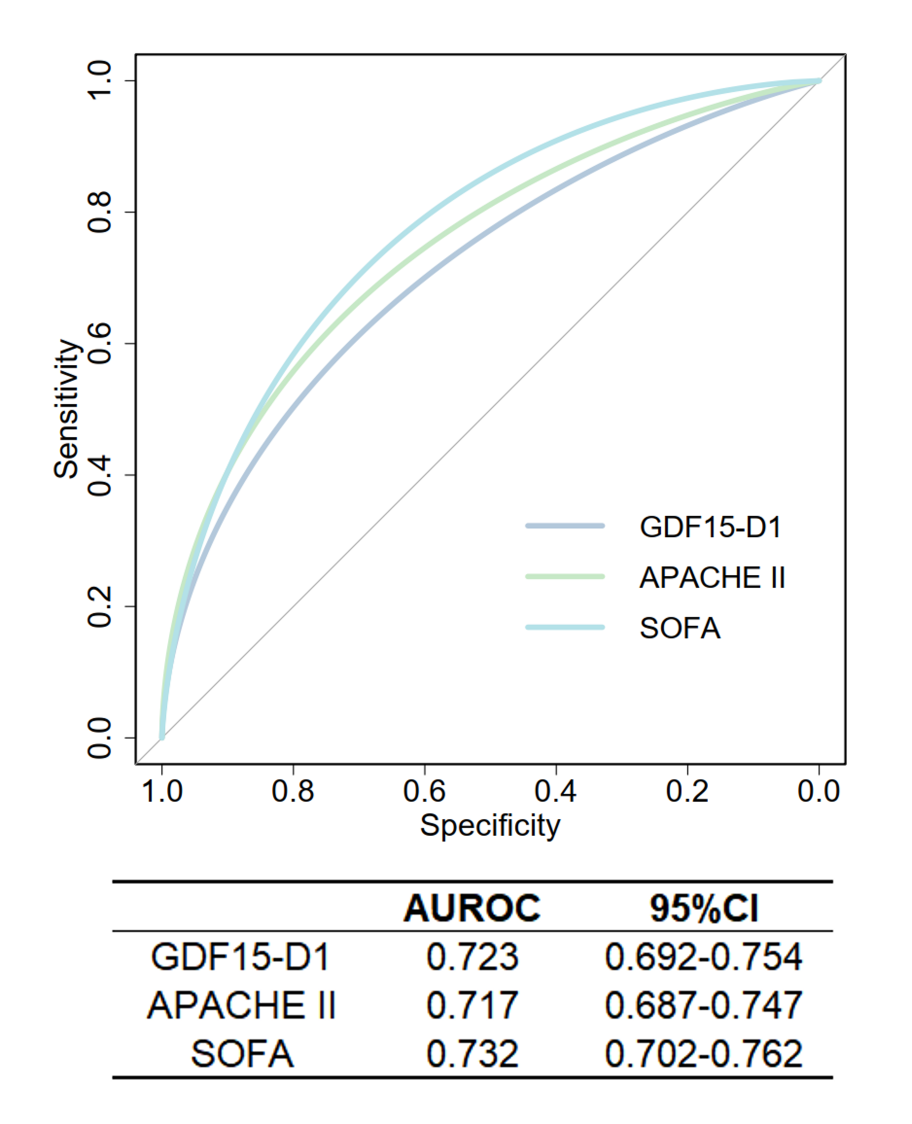

Supplement: Supplemental Information 2 [file peerj-13-20317-s002.png]

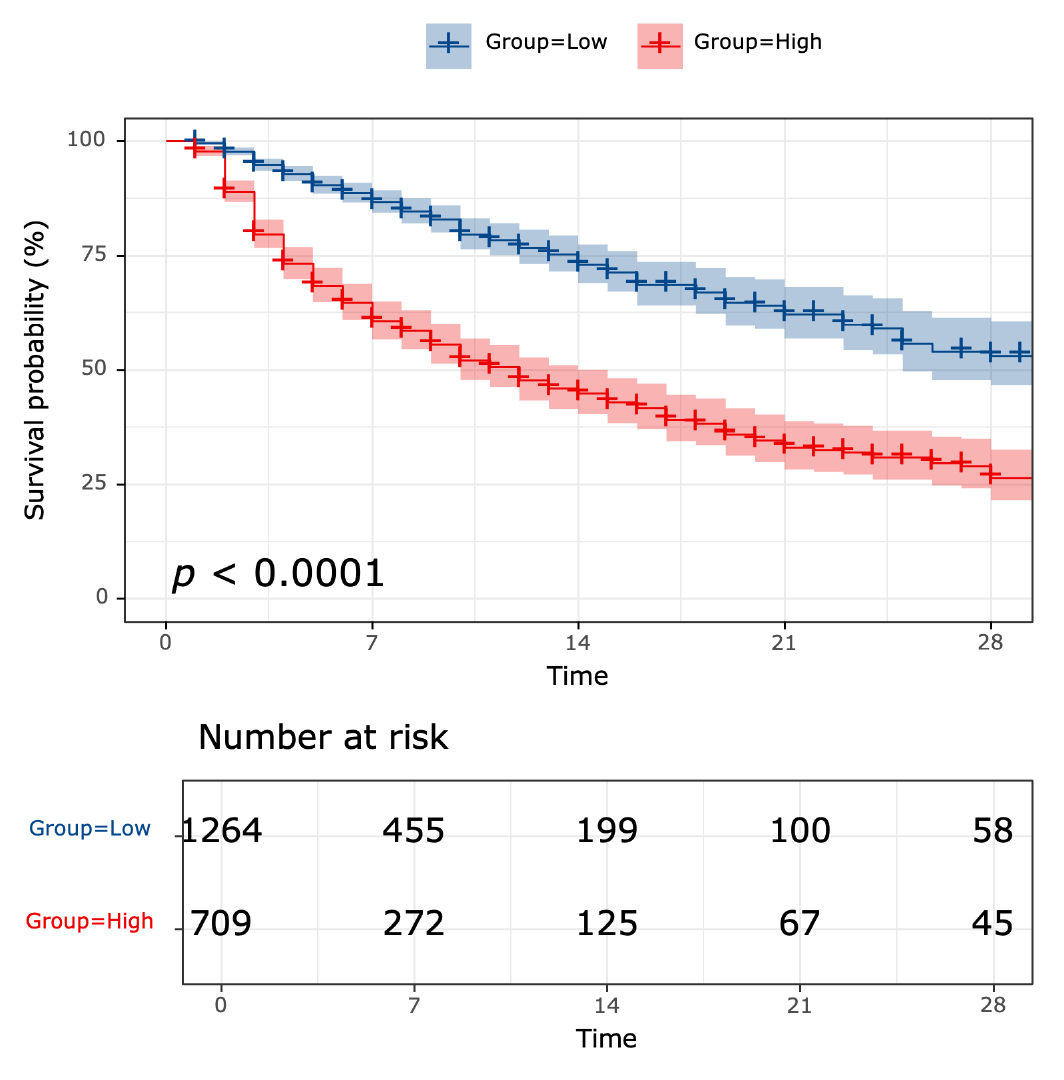

Supplement: Supplemental Information 3 [file peerj-13-20317-s003.png]
