# Supplementary material for: Serum growth differentiation factor 15 trajectory predicts 28-day mortality in critically ill patients: a multicenter cohort study
Source: PeerJ. 2025 Nov 3;13:e20317. doi: 10.7717/peerj.20317 (PMC12591050; doi:10.7717/peerj.20317)
Supplement: Supplemental Information 4 [file peerj-13-20317-s004.docx]

**Table S1: Baseline characteristics of study participants**

| **Characteristic** | **Overall**  **N = 1,973** | **Development Cohort**  **N = 493** | **Validation Cohort**  **N = 1,480** | ***p*-value** |
| --- | --- | --- | --- | --- |
| **Sex [n, (%)]** |  |  |  | 0.013^*^ |
| Male | 1,244 (63.1) | 334 (68.0) | 910 (61.5) |  |
| Female | 729 (36.9) | 159 (32.3) | 570 (38.5) |  |
| **Age (years) [Median, (Q1, Q3)]** | 61 (51, 71) | 60 (50, 71) | 61 (51, 71) | 0.3 |
| **Reasons for ICU Admission [n, (%)]** |  |  |  | < 0.001^*^ |
| Postoperative | 633 (32.1) | 103 (20.9) | 530 (35.8) |  |
| Physical disease | 1,014 (51.4) | 305 (61.9) | 709 (47.9) |  |
| Severe injury | 203 (10.3) | 57 (11.6) | 146 (9.9) |  |
| Malignant tumor | 97 (4.9) | 25 (5.1) | 72 (4.9) |  |
| Other | 26 (1.3) | 3 (0.6) | 23 (1.6) |  |
| **Clinical Comorbidities** **[n, (%)]** |  |  |  |  |
| Hypertension | 498 (25.2) | 119 (24.1) | 379 (25.6) | 0.5 |
| Diabetes | 342 (17.3) | 76 (15.4) | 266 (18.0) | 0.2 |
| Chronic heart failure | 455 (23.1) | 98 (19.9) | 357 (24.1) | 0.051 |
| Chronic hepatic insufficiency | 227 (11.5) | 61 (12.4) | 166 (11.2) | 0.5 |
| Chronic renal insufficiency | 447 (22.7) | 111 (22.5) | 336 (22.7) | 0.9 |
| **Serum GDF15 levels** **(pg/mL)**  **[Median, (Q1, Q3)]** |  |  |  |  |
| GDF15-D1 | 6,307 (3,300, 10,945) | 6,188 (3,287, 11,214) | 6,370 (3,306, 10,631) | > 0.9 |
| GDF15-D3 | 6,033 (3,405, 10,567) | 6,033 (3,409, 10,642) | NA |  |
| GDF15-D7 | 6,562 (3,393, 12,121) | 6,562 (3,393, 12,121) | NA |  |
| **Laboratory tests**  **[Median, (Q1, Q3)]** |  |  |  |  |
| WBC (×10^9^/L) | 11 (8, 15) | 12 (8, 15) | 11 (8, 15) | 0.4 |
| PCT (ng/mL) | 1 (0, 4) | 1 (0, 4) | 0 (0, 4) | 0.078 |
| CRP (mg/L) | 44 (20, 82) | 60 (16, 82) | 44 (16, 82) | < 0.001^*^ |
| pro-BNP (pg/mL) | 968 (211, 3,903) | 945 (223, 3,903) | 974 (207, 3,903) | > 0.9 |
| ALT (U/L) | 17 (7, 36) | 20 (10, 38) | 16 (7, 36) | < 0.001^*^ |
| AST (U/L) | 33 (21, 60) | 33 (21, 61) | 33 (21, 59) | 0.3 |
| TBil (μmol/L) | 17 (11, 28) | 16 (11, 28) | 17 (11, 28) | 0.7 |
| SCr (μmol/L) | 91 (65, 154) | 85 (61, 141) | 93 (67, 154) | 0.006^*^ |
| BUN (mmol/L) | 10 (6, 13) | 9 (6, 13) | 10 (6, 13) | 0.5 |
| APTT (s) | 33 (29, 39) | 32 (28, 38) | 33 (30, 39) | 0.002^*^ |
| Lac (mmol/L) | 1.6 (1.0, 2.5) | 1.6 (1.1, 2.6) | 1.6 (1.0, 2.4) | 0.4 |
| **Critical Care Scores**  **[Median, (Q1, Q3)]** |  |  |  |  |
| APACHE II | 18 (15, 21) | 19 (15, 22) | 18 (15, 21) | 0.021^*^ |
| SOFA | 7 (5, 9) | 8 (5, 10) | 7 (6, 9) | 0.001^*^ |
| **28-day Mortality** **[n, (%)]** | 510 (25.8) | 138 (27.9) | 372 (25.1) | 0.2 |

**Abbreviations:** ICU, intensive care unit; GDF15, growth differentiation factor 15; WBC, white blood cell count; PCT, procalcitonin; CRP, C-reactive protein; pro-BNP, pro-B-type natriuretic peptide; ALT, alanine aminotransferase; AST, aspartate aminotransferase; TBil, total bilirubin; SCr, serum creatinine; BUN: blood urea nitrogen; APTT, activated partial thromboplastin time; Lac, lactate; APACHE II, Acute Physiology and Chronic Health Evaluation II; SOFA, Sequential Organ Failure Assessment; NA, not available.

* *p* < 0.05, significant statistical difference.
