# Supplementary material for: Serum growth differentiation factor 15 trajectory predicts 28-day mortality in critically ill patients: a multicenter cohort study
Source: PeerJ. 2025 Nov 3;13:e20317. doi: 10.7717/peerj.20317 (PMC12591050; doi:10.7717/peerj.20317)
Supplement: Supplemental Information 5 [file peerj-13-20317-s005.docx]

**Table S2: Model fit evaluation for GBTM-based serum GDF15 level trajectory analysis in ICU patients**

| **Group** | **AIC** | **BIC** | **SABIC** | **entropy** | **%class1** | **%class2** | **%class3** | **%class4** | **%class5** |
| --- | --- | --- | --- | --- | --- | --- | --- | --- | --- |
| **2** | 28846.86 | 28909.87 | 28862.26 | 0.80 | 47.9 | 52.1 |  |  |  |
| **3** | 28714.32 | 28798.33 | 28734.85 | 0.72 | 27.2 | 28.4 | 44.4 |  |  |
| **4** | 28658.18 | 28763.20 | 28683.85 | 0.71 | 14.6 | 29.4 | 16.8 | 39.2 |  |
| **5** | 28734.60 | 28860.62 | 28765.39 | 0.70 | 27.2 | 0.00 | 42.0 | 30.8 | 0.00 |

**Abbreviations:** AIC, Akaike information criterion; BIC, Bayesian information criterion; SABIC, sample-size adjusted Bayesian information criterion.
