# Supplementary material for: Serum growth differentiation factor 15 trajectory predicts 28-day mortality in critically ill patients: a multicenter cohort study
Source: PeerJ. 2025 Nov 3;13:e20317. doi: 10.7717/peerj.20317 (PMC12591050; doi:10.7717/peerj.20317)
Supplement: Supplemental Information 6 [file peerj-13-20317-s006.docx]

| **Groups** | **Total (n)**  **N = 493** | **Survival [n (%)]**  **N = 355** | **Death [n (%)]**  **N = 138** | **χ²** | ***p*-value** |
| --- | --- | --- | --- | --- | --- |
| **LM** | 72 | 64 (88.9) ^a^ | 8 (11.1) ^a^ | 48.47 | < 0.001^*^ |
| **MM** | 145 | 125 (86.2) ^a^ | 20 (13.8) ^a^ |  |  |
| **HI** | 193 | 122 (63.2) ^b^ | 71 (36.8) ^b^ |  |  |
| **HP** | 83 | 44 (53.0) ^b^ | 39 (47.0) ^b^ |  |  |

**Table S3: Comparison of 28-day mortality rates among GDF15 trajectory subtypes.**

**Abbreviations:** LM, low-maintenance group; MM, medium-maintenance group; HI, high-increase group; HP, high-persistent group.

* *p* < 0.05, significantly different.

^a^ Chi-square test showed no statistically significant difference between the LM and MM group.

^b^ Chi-square test showed no statistically significant difference between the HI and HP group.
