# Supplementary material for: Serum growth differentiation factor 15 trajectory predicts 28-day mortality in critically ill patients: a multicenter cohort study
Source: PeerJ. 2025 Nov 3;13:e20317. doi: 10.7717/peerj.20317 (PMC12591050; doi:10.7717/peerj.20317)
Supplement: Supplemental Information 7 [file peerj-13-20317-s007.docx]

**Table S4: Multivariate logistic regression model of predictors for 28-day mortality in the validation cohort (n = 1,480)**

| **Characteristic** | **OR** | **95% CI** | ***p*-value** |
| --- | --- | --- | --- |
| Sex | 0.760 | 0.554 – 1.042 | 0.088 |
| Age | 1.022 | 1.012 - 1.033 | 0.000^*^ |
| GDF15-D1^a^ | 1.115 | 1.089 - 1.141 | 0.000^*^ |
| PCT | 1.004 | 1.000 - 1.008 | 0.081 |
| CRP | 1.005 | 1.003 - 1.007 | 0.000^*^ |
| pro-BNP | 0.996 | 0.973 - 1.019 | 0.717 |
| ALT | 0.998 | 0.996 – 1.000 | 0.012^*^ |
| AST | 1.001 | 1.000 - 1.002 | 0.015^*^ |
| TBil | 1.001 | 0.998 - 1.003 | 0.552 |
| SCr | 0.999 | 0.998 - 1.000 | 0.119 |
| BUN | 1.010 | 0.999 - 1.021 | 0.075 |
| Lac | 1.073 | 1.006 - 1.145 | 0.031^*^ |
| APACHE II | 1.110 | 1.081 - 1.139 | 0.000^*^ |
| SOFA | 1.240 | 1.176 - 1.307 | 0.000^*^ |

**Abbreviations:** OR, odds ratio; CI, confidence interval; GDF15, growth differentiation factor 15; PCT, procalcitonin; CRP, C-reactive protein; pro-BNP, pro-B-type natriuretic peptide; ALT, alanine aminotransferase; AST, aspartate aminotransferase; TBil, total bilirubin; SCr, serum creatinine; BUN: blood urea nitrogen; Lac, lactate; APACHE II, Acute Physiology and Chronic Health Evaluation II; SOFA, Sequential Organ Failure Assessment.

^a^ GDF15-D1 was measured in ng/mL.

* *p* < 0.05, significant statistical difference.
