# Supplementary material for: Serum growth differentiation factor 15 trajectory predicts 28-day mortality in critically ill patients: a multicenter cohort study
Source: PeerJ. 2025 Nov 3;13:e20317. doi: 10.7717/peerj.20317 (PMC12591050; doi:10.7717/peerj.20317)
Supplement: Supplemental Information 8 [file peerj-13-20317-s008.docx]

**Table S5: Clinical characteristics and differential analysis of postoperative ICU patient in the validation cohort**

| **Characteristic** | **Overall**  **N = 529** | **Survival**  **N = 432** | **Death**  **N = 97** | ***p*-value** |
| --- | --- | --- | --- | --- |
| **Sex [n, (%)]** |  |  |  | 0.004^*^ |
| Male | 330 (62.4) | 257 (59.5) | 73 (75.3) |  |
| Female | 199 (37.6) | 175 (40.0) | 24 (26.1) |  |
| **Age (years) [Median, (Q1, Q3)]** | 59 (51, 68) | 59 (51, 66) | 61 (53, 71) | 0.037^*^ |
| **Clinical Comorbidities** **[n, (%)]** |  |  |  |  |
| Hypertension | 115 (21.7) | 97 (22.2) | 18 (19.6) | 0.4 |
| Diabetes | 56 (10.6) | 47 (10.9) | 9 (9.3) | 0.6 |
| Chronic heart failure | 135 (25.5) | 111(25.7) | 24 (24.7) | 0.8 |
| Chronic hepatic insufficiency | 29 (5.5) | 23 (5.3) | 6 (6.2) | 0.7 |
| Chronic renal insufficiency | 50 (9.5) | 34 (7.9) | 16 (16.5) | 0.009^*^ |
| **Surgical Site [n, (%)]** |  |  |  | 0.011^*^ |
| Brain | 129 (24.4) | 97 (22.5) | 32 (33.0) |  |
| Bone | 44 (8.3) | 33 (7.6) | 11 (11.3) |  |
| Alimentary canal | 118 (22.3) | 102 (23.6) | 16 (16.5) |  |
| Cardiovascular system | 181 (34.2) | 158 (36.6) | 23 (23.7) |  |
| Other | 57 (10.8) | 42 (9.7) | 15 (15.5) |  |
| **Serum GDF15 levels** **(pg/mL)**  **[Median, (Q1, Q3)]** |  |  |  |  |
| GDF15-D1 | 6,048  (3,262, 10,251) | 5,386  (3,033, 8,511) | 12,952  (6,307, 18,387) | < 0.001^*^ |
| **Laboratory tests**  **[Median, (Q1, Q3)]** |  |  |  |  |
| WBC (×10^9^/L) | 12 (9, 15) | 12 (9, 15) | 12 (8, 16) | 0.7 |
| PCT (ng/mL) | 0 (0, 2) | 0 (0, 2) | 1 (0, 6) | < 0.001^*^ |
| CRP (mg/L) | 20 (6, 57) | 20 (5, 52) | 42 (15, 94) | < 0.001^*^ |
| pro-BNP (pg/mL) | 595 (153, 2,028) | 492 (135, 1,784) | 1,322 (353, 3,903) | < 0.001^*^ |
| ALT (U/L) | 15 (7, 32) | 15 (7, 32) | 16 (8, 29) | 0.6 |
| AST (U/L) | 34 (23, 54) | 33 (22, 53) | 42 (25, 60) | 0.025^*^ |
| TBil (μmol/L) | 18 (12, 28) | 17 (12, 27) | 21 (13, 34) | 0.044^*^ |
| SCr (μmol/L) | 86 (68, 122) | 83 (65, 115) | 111 (79, 164) | < 0.001^*^ |
| BUN (mmol/L) | 9 (6, 13) | 8 (6, 13) | 12 (7, 13) | 0.002^*^ |
| APTT (s) | 32 (30, 37) | 32 (29, 36) | 33 (30, 40) | 0.028^*^ |
| Lac (mmol/L) | 1.8 (1.2, 2.9) | 1.8 (1.2, 2.7) | 2.2 (1.2, 3.4) | 0.022^*^ |
| **Critical Care Scores**  **[Median, (Q1, Q3)]** |  |  |  |  |
| APACHE II | 16 (12, 21) | 16 (11, 18) | 22 (17, 28) | < 0.001^*^ |
| SOFA | 7 (6, 9) | 6 (5, 9) | 7 (7, 11) | < 0.001^*^ |

**Abbreviations:** GDF15, growth differentiation factor 15; WBC, white blood cell count; PCT, procalcitonin; CRP, C-reactive protein; pro-BNP, pro-B-type natriuretic peptide; ALT, alanine aminotransferase; AST, aspartate aminotransferase; TBil, total bilirubin; SCr, serum creatinine; BUN: blood urea nitrogen; APTT, activated partial thromboplastin time; Lac, lactate; APACHE II, Acute Physiology and Chronic Health Evaluation II; SOFA, Sequential Organ Failure Assessment.

* *p* < 0.05, significantly different by ANOVA or Kruskal Wallis test.
