# Supplementary material for: Serum growth differentiation factor 15 trajectory predicts 28-day mortality in critically ill patients: a multicenter cohort study
Source: PeerJ. 2025 Nov 3;13:e20317. doi: 10.7717/peerj.20317 (PMC12591050; doi:10.7717/peerj.20317)
Supplement: Supplemental Information 9 [file peerj-13-20317-s009.docx]

**Table S6: Multivariate logistic regression model of predictors for 28-day mortality in postoperative ICU patients (n = 529)**

| **Characteristic** | **OR** | **95% CI** | ***p*-value** |
| --- | --- | --- | --- |
| Sex | 0.573 | 0.312 – 1.051 | 0.072 |
| Age | 1.024 | 1.004 - 1.045 | 0.019^*^ |
| GDF15-D1^a^ | 1.130 | 1.086 – 1.174 | 0.000^*^ |
| PCT | 1.000 | 0.989 - 1.012 | 0.942 |
| CRP | 1.005 | 1.001 - 1.009 | 0.007^*^ |
| SCr | 1.001 | 0.999- 1.003 | 0.614 |
| Lac | 1.100 | 0.987 - 1.226 | 0.084 |
| APACHE II | 1.104 | 1.063 - 1.145 | 0.000^*^ |
| SOFA | 1.159 | 1.064 - 1.262 | 0.001^*^ |

**Abbreviations:** OR, odds ratio; CI, confidence interval; GDF15, growth differentiation factor 15; PCT, procalcitonin; CRP, C-reactive protein; SCr, serum creatinine; Lac, lactate; APACHE II, Acute Physiology and Chronic Health Evaluation II; SOFA, Sequential Organ Failure Assessment.

^a^ GDF15-D1 was measured in ng/mL.

* *p* < 0.05, significant statistical difference.
