# Supplementary material for: Serum growth differentiation factor 15 trajectory predicts 28-day mortality in critically ill patients: a multicenter cohort study
Source: PeerJ. 2025 Nov 3;13:e20317. doi: 10.7717/peerj.20317 (PMC12591050; doi:10.7717/peerj.20317)
Supplement: Supplemental Information 12 [file peerj-13-20317-s012.docx]

Supplemental File: We Provide a codebook to convert numbers to their respective factors

**Sex**: 0 = female, 1 = male;

**28-day survival status**: 0 = Survival, 1 = Death;

**Development/Validation Cohort**: 0 = Development Cohort, 1 = Validation Cohort;

**Postoperative group**: 0 = Non-postoperative group, 1 = Non-postoperative group;

**Reasons for ICU admission**: 1 = Postoperative, 2 = Physical disease, 3 = Severe injury,

4 = Malignant tumor, 5 = Other;

**Hypertension**:0 = Non-hypertension, 1 = Hypertension;

**Diabetes**: 0 = Non-Diabetes, 1= Diabetes;

**Chronic heart failure**:0 = Non-Chronic heart failure, 1 = Chronic heart failure

**Chronic hepatic insufficiency**:0 = Non-Chronic hepatic insufficiency, 1 = Chronic hepatic insufficiency;

**Chronic renal insufficiency**:0 = Non- Chronic renal insufficiency, 1 = Chronic renal insufficiency;

**Surgical site**:1 = Brain, 2 = Bone, 3 = Alimentary canal, 4 = Cardiovascular system, 5 = Other;

**Traj_Group**:1 = LM, 2 = MM, 3 = HD, 4 = HP.
